# Investigation on the Aggregation Behaviors and Filament Morphology of Tau Protein by a Simple 90° Angle Light-Scattering Assay

**DOI:** 10.1155/2013/354730

**Published:** 2013-09-15

**Authors:** Hai-Lin Liao, Ling-Feng Jiang, Tian-Ming Yao

**Affiliations:** ^1^Faculty of Pharmacy, Guangxi University of Chinese Medicine, P. O. Box 6, 179 Mingxiudong Road, Nanning, Guangxi 530001, China; ^2^Department of Chemistry, Tongji University, Shanghai 200092, China

## Abstract

The *in vitro* aggregation of tau constructs was monitored by a simple 90° angle light-scattering (LS) approach which was conducted directly on fluorescence instrument. At the optimum incident wavelength (550 nm, unpolarized), the sensitivity of LS was high enough to detect tau aggregation at micromolar range. The nucleation and elongation, different events in the aggregation process of 4RMBD construct (corresponding with the four repeated units of tau Microtubule Binding Domain) could be observed by this approach, as compared with ThS fluorescence assay. The validity of this technique was demonstrated over a range of tau concentrations with different tau filaments. Linear regression of scattering light against concentration yielded the *x*-intercept, the critical concentrations of tau constructs. The critical concentrations of 4RMBD and its S305N mutant are 5.26 *μ*M and 4.04 *μ*M respectively, indicating point mutation S305N, which is associated with FTDP-17, appear to enhance the heparin-induced tau aggregation *in vitro*. Furthermore, the slopes of concentration dependence curves, as well as the angle dependence, were discussed based on the filaments morphology examined by electron microscopy and ultrasonication experiment.

## 1. Introduction

Microtubule-associated protein tau is a highly soluble protein that shows hardly any tendency to assemble under physiological conditions. In the brains of Alzheimer's disease (AD) patients; however, tau dissociates from the axonal microtubule and abnormally aggregate to form paired helical filaments (PHFs) [1]. It is therefore of some importance to understand which part and what changes in the tau molecule can result in its transformation into a pathological entity, as such knowledge could potentially lead to the development of therapeutic strategies.

Eleven exons make up the longest human brain tau isoform. With expressed regions 2, 3, and 10 being subject to alternative mRNA splicing, the six tau isoforms expressed in adult human brain range from 352 to 441 amino acids in length. Recent investigations demonstrated that the repeat units in microtubule-binding domain (MBD) prove to be the core structure for tau microtubule association function and also PHF assembly [2, 3]. Although all the repeat units contain 31~32 amino acids and their amino sequences are similar, their affinities to bind with microtubule and their roles in PHF formation, are assorted. Additionally, the repeat units could be separated into two regions, a highly conserved 18 amino acid sequence and a less conserved 13 amino acid sequence. Using the peptides (18 residues) corresponding to the conserved sequences of the four repeats, Arrasate et al. reported a different aggregation behavior for each repeat, while neglecting the effect of the less conserved region [4]. We previously investigated the* in vitro *aggregation features of tau peptides R1, R2, R3, and R4 (31~32 amino acids) corresponding to the first, to the fourth repeat unit respectively, clarified the possible role of each repeat structure in filament formation [3]. 

For monitoring the *in vitro* aggregation of tau, different techniques have been employed. They are (i) sedimentation assays [4], (ii) qualitative and quantitative electron microscopy [5, 6], and (iii) intrinsic and extrinsic fluorescence [7, 8]. 

The intrinsic fluorescence could be used to probe the conformation changes of tau protein [8]. However, the primary structures of most tau constructs do not contain any Try residues. Usually a recombinant tau containing Try point mutation in peptide chain was used in this method. For extrinsic fluorescence method, an organic dye is introduced as fluorescent probe. It has been reported that tau polymerization can be monitored by measuring the increase in thioflavin S (ThS) fluorescence in the presence of tau polymers in real time [7]. This technique has shown promise, but the minimum unit of tau polymer responsible for the increase in fluorescence of ThS is still unknown. This raises the risk factor that aggregates, but not necessarily tau filaments, are capable of binding to ThS, and that interpretation of the resulting data could be misleading. Similarly, while electron microscopy is a valuable tool for confirming the presence of tau filaments and elucidating their structure, it is insufficient for mechanistic analysis of tau polymerization in which numerous variables should be tested. These points have led many researchers to use EM as a qualitative tool only [5, 6]. 

A widely used method to assess the polymerization of filamentous macromolecular compounds is to measure the turbidity of the reaction solution by means of “light scattering” [9]. However, turbidimetric analysis requires that enough filaments be formed to cause a decrease in the amount of light transmitted through a protein solution, and this technique has not proven sensitive enough to monitor tau aggregation at physiological condition (around 4 *μ*M). A 90° light scattering method was used by Mukherjee and Lutkenhaus to monitor the assembly of FtsZ, an ancestral homologue of tubulin [10], and this method was shown to be more sensitive than conventional turbidity measurement. Gamblin et al. successfully employed the laser light scattering (LLS) method to measure the *in vitro* polymerization of tau protein [11]. They increased the sensitivity of light scattering by employing an argon laser beam (*λ* = 488 nm) as the incident light and a digital camera to captured the scattered light at an angle of 90°. Unsatisfactorily, this method required a special laser/optical system which was inconvenient to organize by other laboratories. 

This led us to investigate the utility of a simple 90° angle light scattering (LS) approach in monitoring the *in vitro* fibrillization of tau protein. Some interesting results regarding the different aggregation behaviors of tau peptides R1~R4 have been reported earlier [3]. In the present work, the tau construct, 4RMBD, and its mutant S305N, corresponding to the whole microtubule-binding domain, as well as the tau peptide, R3 (31 residue), corresponding to the third repeat segment (306~336), were synthesized. The goal of this paper is to demonstrate the validity of this technique systematically, with the fibrilliations of different tau constructs at micromolar range. Some factors regulating the intensity of scattering light have been discussed based on the theoretical principle. By analyzing the effect of filament morphology, we have correlated the scattering light with the filament concentration. Furthermore, using this method we have found that the critical concentrations of 4RMBD S305N mutant are lower than its wild type, indicating S305N mutant associated with FTDP-17 appears to enhance the heparin-induced aggregation. 

The light scattering assay described herein avoids many of the pitfalls associated with LLS and ThS binding. Compared with LLS, our method has equal high sensitivity, although the incident beam is not polarized; moreover, it can be performed directly on fluorescence instrument and is easy to use. Unlike ThS florescence methods, this approach does not introduce any external dye as “molecular probe”, and therefore exerting no disturbance on the aggregation reaction. 

## 2. Materials and Methods 

### 2.1. Chemicals and Tau Peptide

**Figure 1:**
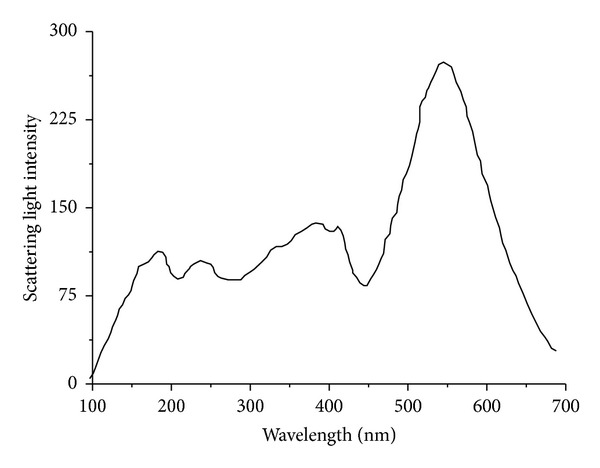
Light scattering spectrum of filamentous tau solution (10 *μ*M 4RMBD incubated at 37°C for 5 hr. by the aggregation inducing reagent, heparin).

Heparin (average molecular weight (MW), 6000), ThS were obtained from Sigma. Tau peptide R3 was purchased from American Peptide Company, from which synthetic details can be obtained upon request. The peptide (including TFA as counter ion) was obtained in the lyophilized form, and the purity was determined to be >95.0% by reverse-phase HPLC. Working solution of tau peptide was made by dilution to 1 mg/mL with 50 mM Tris-HCl buffer (pH 7.5) immediately before use. The expression and purification of His-tagged four-repeat 4RMBD of human brain tau (Figure 1) were performed according to a previous paper [12]. The purity was confirmed by SDS-PAGE analysis.

### 2.2. Light Scattering Assay for Tau Peptide Aggregation

The aggregation of tau peptide was measured by 90° angle light scattering in a JASCO spectrofluorometer (model FP6500) with both excitation and emission wavelengths set at 550 nm and a slit width of 3 nm. Tau peptide was added to a final concentration of 0.5 *μ*g/mL (15 *μ*M) or as specified in the 50 mM Tris-HCl buffer to a fluorometer cuvette with a 1-cm path length. The cuvette was then placed in a cuvette chamber that was maintained at 37°C by a circulating water bath, and data were collected for 8 min to establish a baseline. Then the cuvette was removed, and 3 *μ*M heparin was added to achieve a final reaction volume of 300 *μ*L. The reaction mixture was gently stirred with a pipette tip, and the cuvette was returned to the cuvette chamber for data collection for a specified period of time. The reading at time zero is the first reading taken after the cuvette was returned to the chamber following heparin addition. The elapsed time for the heparin addition step was 20 to 30 s. The net change in light scattering following heparin addition was plotted as a function of time. Although data were collected every 2 s, for purposes of plotting, we used only data obtained every 30 s or 1 min and in some instances 2 min.

### 2.3. Monitor of Aggregation by Fluorescence Measurement

Tau peptide was adjusted to a concentration of 15 *μ*M using 50 mM Tris-HCl buffer (pH 7.5) containing 10 *μ*M ThS dye. The aggregation was induced by adding heparin to the solution (final concentration was 3.8 *μ*M) and mixing with a pipette prior to fluorescence measurement. The time scan of the fluorescence was carried out on a JASCO FP-770F instrument with a 2-mm quartz cell, where the temperature was kept at 37°C by a circulating water bath. The aggregation kinetics was analyzed by recording the time-dependent curve of the fluorescence intensity with excitation at 440 nm and emission at 490 nm. The excitation and emission slit widths were set at 10 nm. Background fluorescence of the sample was subtracted when needed.

### 2.4. Electron Microscopy

Fifteen *μ*M tau peptide was mixed with 3.8 *μ*M heparin in 50 mM Tris-HCl pH 7.5 buffer. The solution was then incubated at 37°C for 100 min. The 600-mesh copper grids were used for the negative staining EM. The grids were placed with a drop of the protein solution and a drop of 2% uranyl acetate. After 2 min, excess fluid was removed from the grids. Negative staining electron microscopy was performed in a electron microscope (Hitachi H-600) operated at 75 kV.

## 3. Results and Discussion

### 3.1. The Optimum Wavelength of Incident Light for Light Scattering on Tau Filaments

A conventional light scattering method to assess the polymerization of filamentous macromolecular compounds is the turbidimetric analysis of the solution following the induction of polymerization [13]. However, we found that the turbidity measurement by a spectrophotometer was not sensitive enough to detect tau aggregation at the physiological level (1 *μ*M~5 *μ*M). This prompted us to seek a method with higher sensitivity for tau fibrillization investigation. The 90° angle light scattering assay directly on a fluorometer came to our notice, since such an assay had been used to monitor the assembly of FtsZ [10], similar to the case of tau aggregation. If it succeeded, it could be a powerful tool for tau pathology due to its conveniences.

The intensity of scattering light is theoretically dependent on *λ*, the wavelength of incident light. It has been reported that 90° angle light scattering, at the incident light *λ* = 350 nm, could be used to monitor FtsZ polymerization [10] while laser light scattering (LLS), at the polarized incident light *λ* = 488 nm, could be used to study tau polymerization [11]. In order to determine the optimum wavelength of incident light with respect to the dimensions of tau filaments, a light scattering spectrum of the filamentous tau solution was recorded. As shown in Figure 1, the intensity of scattering light reaches its maximum at the wavelength of 550 nm. Accordingly, in the subsequent experiment, the 90° angle light scattering assay for monitoring tau polymerization was conducted on a fluorometer with both excitation and the emission wavelengths fixed at 550 nm.

### 3.2. Different Events in the Process of Aggregation Can Be Observed by Comparing Light Scattering Assay with ThS Fluorescence Method

**Figure 2:**
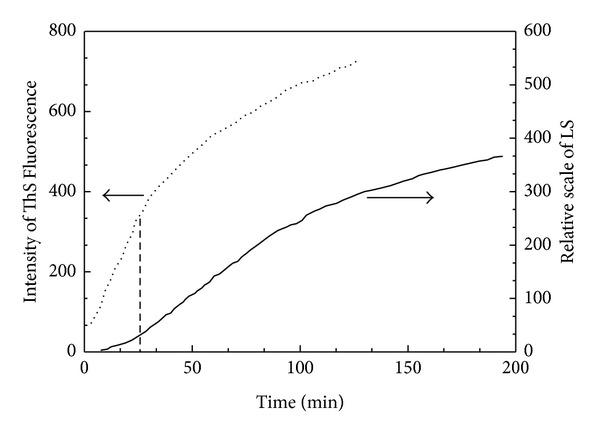
Comparison of light scattering (solid line) with ThS fluorescence assay (dotted line) for monitoring the accumulation of 4RMBD.

As an example, the light scattering kinetic curve (solid line) of the fibrillization of 4RMBD was recorded, as shown in Figure 2. Another method commonly used for monitoring tau aggregation is the ThS fluorescence assay. As a comparison, the ThS fluorescence kinetic curve (dotted line) of the fibrillization of 4RMBD is also shown in Figure 2. Both methods can be applied to monitor tau aggregation; however, they are based on different principle, as we discussed in the pervious work [3]. Accordingly, their time-dependence profiles, especially in the beginning period of aggregation, are differently shaped. The light scattering curve shows a characteristic lag period (0~25 min), followed by a period of exponential growth and an asymptotic approach to equilibrium. On the contrary, the lag period of ThS fluorescence curve is indistinct. The minimum unit that is detectable by light scattering is thought to be mature filament, the tau filament with dimensions comparable to the wavelength of incident light. Therefore, in the lag period, the scattering light does not respond to the growth of immature filament which can be detected by ThS fluorescence. As we suggested in the earlier paper, the filament formation was a complicated process which could be divided into several steps, that is, (i) activation, (ii) nucleation and (iii) elongation [14]. In the present work, the nucleation and elongation, different events in aggregation process can be clearly observed by comparing light scattering assay with ThS fluorescence method. The lag period of light scattering curve is probably an indication of the nucleation process of tau aggregation. In the case of 4RMBD, the nucleation step accomplished within about 20 min.

### 3.3. The Concentration Dependence of Light Scattering and the Critical Concentration of Different Tau Peptides

Tau peptide of various concentrations with 3.8 *μ*M heparin in 50 mM Tris-HCl buffer was incubated at 37°C overnight. The intensity of scattering light at 550 nm (*I*_LS_) was plotted against the tau concentration. The points were fit to straight line by linear regression. 

**Figure 3:**
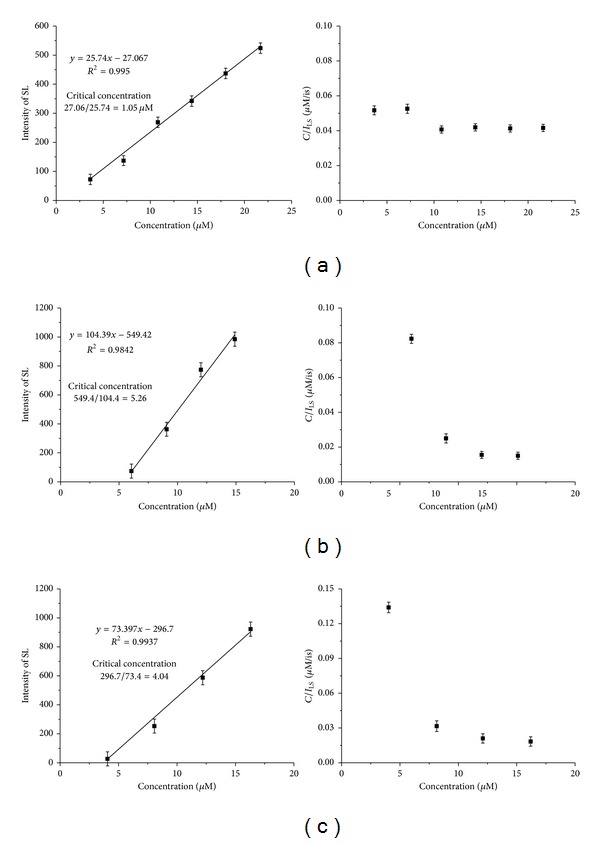
Concentration dependence (left side) and angle dependence (right side) of scattering light of (a) R3 (b) 4RMBD (c) 4RMBD S305N.

As shown in Figure 3, there is a good linear correlation between scattering light and tau concentration, in the range of 3~20 *μ*M, with *R* ^2^ = 0.995 for R3 peptide; 7~15 *μ*M, with *R* ^2^ = 0.984 for 4RMBD wild; and 5~15 *μ*M, with *R* ^2^ = 0.993 for 4RMBD S305N mutant. However, if the concentration is lower than 2 *μ*M, or higher than 20 *μ*M, the linear correlation does not exist because filament is unable to form effectively if the tau concentration is too low, while tau filament may be precipitated from the solution if the concentration is too high.

A very important parameter is the *x*-intercept of the regression line of light scattering. This value indicates the critical concentration, the minimum amount of tau protein that aggregates to form filament. Different from the aggregation rate shown in our earlier report, which reflects the dynamic properties of tau aggregation, the critical concentration is a thermodynamic parameter at steady state. The lower value of critical concentration means that the aggregation proceeds relatively easy, even the concentration of tau solution is low. On the other hand, the higher value implies that the aggregation hardly initiates, unless the concentration of tau solution is high enough.

We have reported that the 4RMBD S305N mutant aggregates more rapidly than 4RMBD wild, as monitored by the ThS fluorescence [12]. In this work, the thermodynamic parameter obtained by LS is consistent with the dynamic data of ThS fluorescence. The critical concentration of 4RMBD S305N mutant (4.04 *μ*M) is lower than that of 4RMBD wild (5.26 *μ*M), indicating that the site mutation promotes the *in vitro* heparin-induced aggregation. It is generally believed that the mutation in the microtubule binding domain plays an important role in PHF formation in frontotemporal dementias FTDP-17 [14–17]. The present data support this assumption and provide a deeper biochemical understanding for the observation that the S305N missense mutation is linked to familial tauopathy [18].

The critical concentration can alternatively be determined by ThS fluorescence assay. For example, plotting the ThS fluorescence against the R3 concentrations (data not shown), the intercept on the *x*-axis yields a critical concentration of 1.96 *μ*M, a little higher than the critical concentration of 1.05 *μ*M determined by light scattering as described herein. However, for the ThS fluorescence approach, the organic dye is introduced into the reaction solution as the molecular probe. We cannot exclude the possibility that ThS dye affects tau aggression, concerning the reports showing organic substances to be inhibitors or promoters of tau polymerization [19, 20]. Besides, the minimum unit of tau polymer responsible for the increase in ThS fluorescence remain to be clarified. In this view, light scattering is more reliable approach for the determination of critical concentration. 

### 3.4. The Angular Dependence of LS

Theoretically, the polarized light scattering by particles, whose diameter are comparable with the wavelength of the incident beam, reduces to the fundamental equation [11, 15] as follows:

(1) $$\begin{array}{c} \frac{K\cdot C}{I_{\text{LS}}}=\left(\frac{1}{P\left(90^{{}^{\circ}}\right)}\right)\cdot \left(\frac{1}{Mw}+2BC\right), \end{array}$$

(2) $$\begin{array}{c} \frac{1}{P\left(90^{{}^{\circ}}\right)}=1+\frac{8\pi^{2}}{3\lambda^{2}}\langle {R_{G}}^{2}\rangle , \end{array}$$

where *K* is a constant, *C *is the concentration of peptide, *I*_LS_ is the intensity of LS, *B* is the second virial coefficient, *Mw* is the weight-averaged molecular weight of the scattering particles, and *P*(90°) is a factor that expresses the angular dependence of LS and reflects the anisotropy of molecular shape. *R*_*G*_ is radius of gyration. When *P*(90°) is postulated to be constant, a plot of *C*/*I*_LS_ versus *C* over a certain concentration range should yield a straight line with a slope proportional to *B* and a *y*-intercept that is inversely proportional to *P*(90°)  *Mw*. 

The plots of *C*/*I*_LS_ *versus C* are also shown in the right side in Figure 3. The *C*/*I*_LS_ values of R3 are almost constant within their experimental errors in the concentration range (Figure 3(a) right side), indicating that the second virial coefficient (*B*) is almost negligible and the filament grows with a constant shape with increasing concentration. In contrast, the plots of *C*/*I*_LS_ *versus C* of 4RMBD wild, as well as its S305N mutant, at the concentration of ≤6 *μ*M (for 4RMBD wild), or ≤4 *μ*M (for 4RMBD S305N mutant), close to or lower than the critical concentration, significantly deviate from their straight lines (Figures 3(b) and 3(c), right side). Since the large *C*/*I*_LS_ value means that the LS intensity per filament particle is weak, this result may imply that the filament of 4RMBD wild (or/and its S305N mutant), formed at a concentration less than 6 *μ*M (or/and 4 *μ*M), is in an immature state for the extension process. In this regard, the critical concentrations of 4RMBD and its S305N mutant also correspond to the minimum concentrations necessary for extending the filament to a mature state (completion of nucleation process). 

### 3.5. The Slope of Concentration Dependence Curve of LS

As exemplified in Figure 3, the slopes of linear regression curves of LS intensity concentration are different from each other; they are 27 *μ*M^−1^ for R3, 104 *μ*M^−1^ for 4RMBD wild, and 73 *μ*M^−1^ for 4RMBD S305N mutant, respectively. This value does not linearly correlate with critical concentration; however, it is likely to be dependent on the morphology of filaments inherent in the respective tau constructs. Although the LS intensity is mainly due to the mass of scattering particles in solution, it is also affected by the shape of the filament because any asymmetry from the spherical shape results in errors based on the Rayleigh scattering theory [16]. Since light scattering had not been frequently applied to tau aggregation, previously, and we felt it necessary to analyze this in some detail.

(1) Rayleigh scattering: for particles that are very small with respect to the wavelength of incident light (*L*/*λ* < 1/50, where *L* is the length of the particle), the scattering lights are uniform in all directions, with intensities proportional to their mass concentration, but inversely proportional to *λ* ^4^.
(2) As the particle dimensions increase to become significant with respect to the wavelength of incident light, but not reached the Berne limit (1/50 < *L*/*λ* < 3.5), the light scattering will deviate from the ideal Rayleigh scattering. The length of the particles could introduce an angular dependence. In other words, decreases in light scattering at 90° could arise from the preferential forward scattering of increasingly long filaments. The deviation from ideal scattering by large particles can be described, in theory, by a term of *P*(90°), as discussed earlier. In this case, a “rigid rod” model is usually adopted. This rigid rod consists of optically isotropic segments distributed uniformly along the rod axis. When unpolarized incident light is scattered from solution containing these rigid rods, the intensity of the light scattered into 90° angle, regardless of the final polarization of the scattering light, is in the Rayleigh-Gans limit [17]. Consider
  (3) $$\begin{array}{c} I\left(q\right)=C\frac{VE_{0}^{2}}{16\pi^{2}R_{0}^{2}{\left(\varepsilon^{\prime }\right)}^{2}}{\left(\frac{2\pi }{\lambda }\right)}^{4}\alpha^{2}S\left(q;L\right), \end{array}$$
 where *C* is the number concentration of rigid rods, *S*(*q*; *L*) is the structure factor of a uniform rigid rod (which is related to its length *L*), *α* is the identical polarizability of optically isotropic segments distributed uniformly along the rod axis, *V* is the scattering volume, *E*_0_ the amplitude of the incident light wave, *R*_0_ is the distance of the detector from the scattering volume, and *ε*′ is the dielectric constant of the medium. Consequently, for “rigid-rod” particles such as tau filaments, the total intensity of scattered light is inevitably affected by the length of the rods.
(3) Berne limit (*L*/*λ* of 3.5): for rod-like particles which are very long with respect to the wavelength of incident light, exceed the Berne limit (*L*/*λ* of 3.5), one can deduce a very important consequence based on (3). That is, the intensity of scattering light is inversely proportional to *λ* ^3^, rather than the usual *λ* ^4^, as observed in Rayleigh scattering. However, the angular dependence of light scattering would not appreciably deviate from some maximum values. In this case, different regions of a single filament could behave as spatially independent scattering units; thus, the scattering lights are dependent on the length of individual scattering units rather than on the filament lengths. Consequently, the intensity of scattering light is directly proportional to the mass concentration.

**Figure 4:**
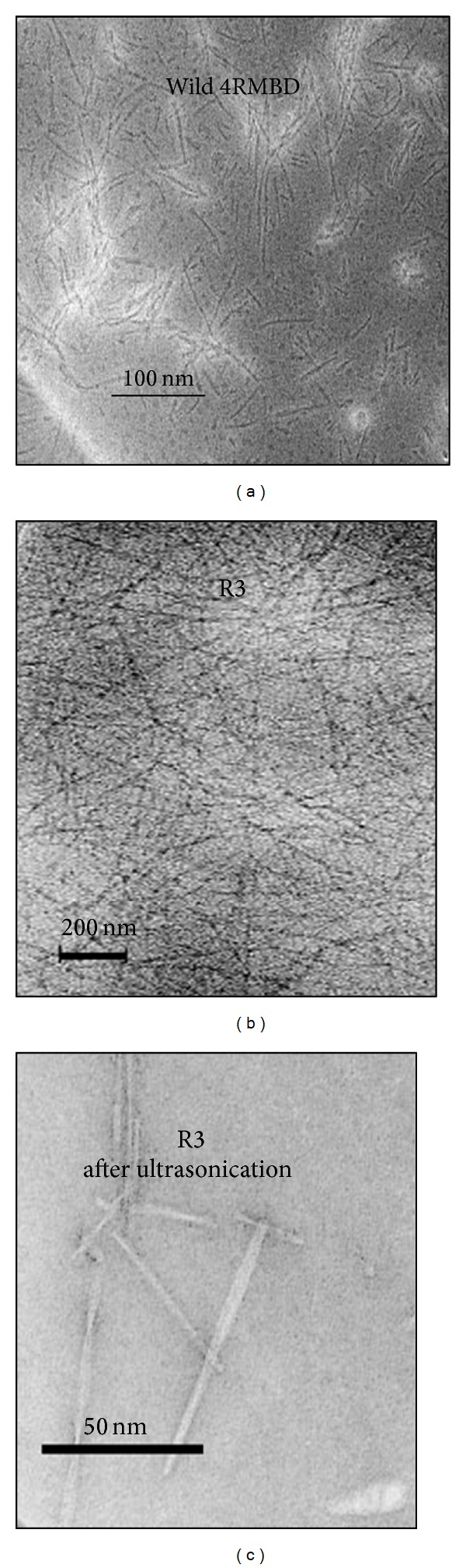
The electronic microscopic picture of different tau filaments (a). The filments of wild 4RMBD (b). The filaments of R3 (c). The filaments of R3 after ultrasonication.

To identify the effect of filament morphology on scattering light, electronic microscopic photos of R3 and 4RMBD filaments were taken. As can be seen in Figure 4, the filament shapes are quite different. Although the paired helical filaments are frequently observed for 4RMBD tau constructs, the filaments of R3 are generally straight. In addition, the R3 filaments (more than 1.5 *μ*m in length) are much longer than 4RMBD filaments (less than 200 nm in length).

Considering the quite different morphology, the difference in the slope of linear regression curves (Figure 3) may be attributed to two aspects. First, tau molecules may be differently arranged in the paired helical filament (4RMBD) and in the straight filament (R3). The value of identical polarizability (*α*) of rod segment, as well as the structure factor *S*(*q*; *L*) in (3), could be different. In other words, the scattering light with respect to each optically isotropic segment could be different. Second, the light scattering belonged to different mode, since the ratio of *L*/*λ* is different. For R3 filament, *L*/*λ* ≈ 3, close to the Berne limit, while for 4RMBD filament, *L*/*λ* < 1, close to the Rayleigh scattering. 

In conclusion, we can correlate the slope of concentration dependence curve with the length of filaments. That is, longer filaments (i.e., R3), which decrease the intensity of scatter light from ideal Rayleigh scattering, will have concentration dependence curve with a smaller slope and with very little angle dependence; while the shorter filaments (i.e., 4RMBD) whose scattering light intensity is close to the Rayleigh scattering, will have concentration dependence curve with a larger slope and with relatively significant angle dependence. 

### 3.6. Effects of Tau Filament Lengths on Light Scattering

Since the electron microscopic photos show that the filaments formed in these experiments have not reached the Berne limit but are, on average, larger than those particles that would exhibit ideal Rayleigh scattering, and we herein carried out an experiment to examine how the filament length affects the intensity of scattering light. 

**Figure 5:**
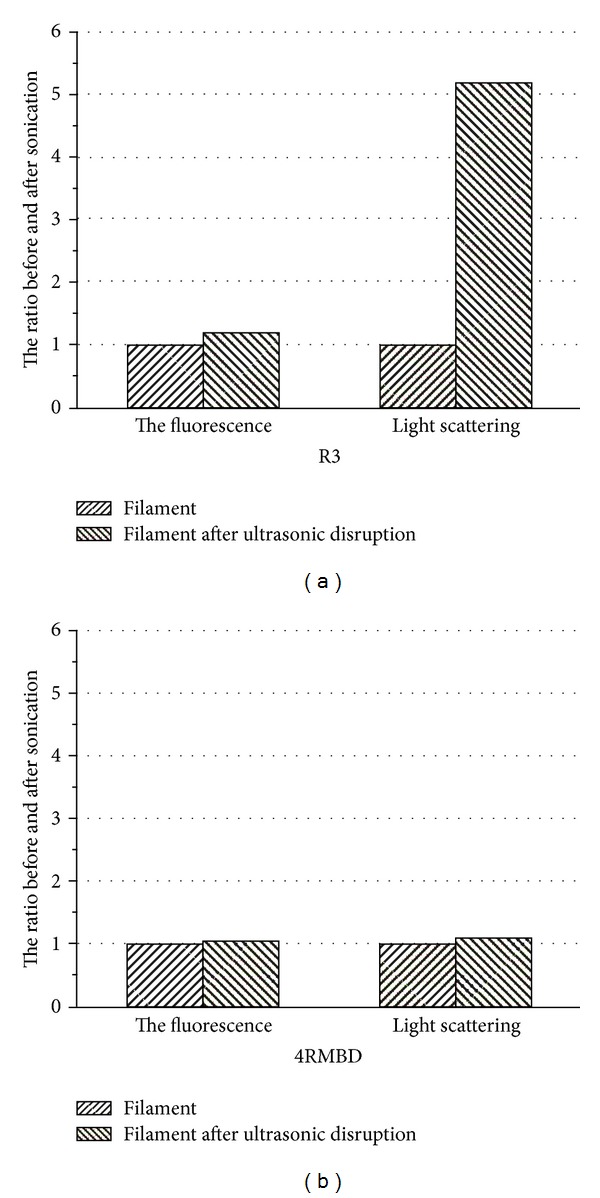
The effect of ultrasonication on light scattering (right side) and ThS fluorescence (left side) of R3 and 4RMBD before (*≡*) and after (///) sonication.

Tau filament of R3 was prepared by adding heparin into the R3 peptide solution and incubated for 8 hrs at 37°C. By ultrasonication, the R3 filament in solution could be disrupted to smaller pieces. As revealed by electron microscopy, the R3 filaments with the average length over 1.5 *μ*m (Figure 4(b)) were broken to the length about 100 nm (Figure 4(c)). The ThS fluorescence and the light scattering intensity before and after ultrasonication were measured as described earlier. The experiment results were illustrated in Figure 5, showing how the ultrasonication affects the scattering light and ThS fluorescence. For both R3 and 4RMBD filaments, there are only slight increases in ThS fluorescence after ultrasonication, probably resulting from the increase of surface area, while the filaments were disrupted into short fragments, and more fluorescence dye was bound on the filament surface. On the other hand, the effects of ultrasonication on light scattering were quite different. For 4RMBD filaments, the change of scattering light intensity was very small when the filaments were disrupted to short pieces by ultrasoniction. This means that the scattering light was almost independent of the length of 4RMBD filaments. The intensity of scattering light was proportional to the mass concentration of filaments. However, for R3 filaments, the scattering light was dramatically increased when the filaments were disrupted to short pieces by ultrasonication (5 times as compared before ultrasonication), indicating that the intensity of scattering light was dramatically affected by the length of R3 filaments. In this case, the intensity of scattering light was dependent on not only the mass concentration of filaments but also the length of filament.

 Therefore, this data of ultrasonication experiments support our suggestion that light scattering of 4RMBD filaments was approximately ideal Rayleigh scattering, while R3 filaments was not, even though for both cases the scattering light was directly proportional to the mass concentration. For 4RMBD filament, *L*/*λ* < 1, the scattering light had already reached its maximum, close to the Rayleigh scattering. Even the filament was broken into shorter spices by ultrasonication, there was no more increase in scattering light. On the other hand, a longer length of R3 filament makes the situation quite different. The most likely cause for this discrepancy lies in two aspects: (i) R3 filaments do not behave as rigid rods (which is a requirement for calculating radius of gyration *R*_*G*_ in (2)). In fact, the electron micrograph (Figure 5) shows that the R3 filaments are curved, suggesting a flexible structure. (ii) The ratio of *L*/*λ* ≈ 3, close to the Berne limit, but much larger than the limit of Rayleigh scattering (*L*/*λ* ≫ 1/50). In that case, different regions of a single filament could behave as spatially independent scattering units which were also larger than the limit of Rayleigh scattering. When the long, flexible filaments were disrupted and broken into a number of small pieces whose size could approach the limit Rayleigh scattering, the total intensity of scattering light was thus increased as the length becomes shorter. 

## 4. Conclusion

It has been shown in this paper that the 90° angle light scattering directly performed on fluorometer is a convenient approach to monitor tau aggregation. Such a common method would greatly enhance future studies in the field of tau aggregation and allow more meaningful comparisons of results obtained in different laboratories. Several conclusions can be drawn from the present data. (1) The intensity of light scattering at 550 nm, regardless of ideal Rayleigh scattering or not, ware proportional to the filament mass as indicated in the concentration dependence curve. Hence, the 90° angle light scattering reflects an accurate estimate of the mass of tau filaments present in the solution. (2) The slope of concentration dependence curve is related to the morphology of tau filaments. That is, longer filaments (i.e., R3) will have a smaller slope while shorter filaments (i.e., 4RMBD) will have a larger slope. (3) The angular dependence became significant with the degree of aggregation of 4RMBD, where the filaments were short and the light scattering was close to the Rayleigh scattering, indicating an immature state at low concentration (less than 6 *μ*M). On the contrary, for the case of R3 aggregation, the angle dependence was insignificant where the filaments were long and the light scattering was approximately at “Berne limit”. (4) Using this method we have found that the critical concentrations of 4RMBD S305N mutant are lower than its wild type, indicating that S305N mutant associated with FTDP-17 appears to enhance the heparin-induced aggregation. 

## Abbreviations

AD: Alzheimer's disease
CD: Circular dichroism
EM: Electron microscopy
LS: Light scattering
LLS: Laser light scattering
MBD: Microtubule-binding domain
4RMBD: Four-repeat microtubule-binding domain
R1 (R2, R3, and R4): First (second, third, and fourth) repeat peptide of the four-repeat microtubule-binding domain
PHF: Paired helical filament
SDS-PAGE: Sodium dodecylsulfate polyacrylamide gel electrophoresis
ThS: Thioflavin S
FTDP-17: Frontotemporal dementia with parkinsonism linked to chromosome 17
FtsZ: Filamentation temperature-sensitive protein Z.
